# Synchronous double primary small cell lung cancer and invasive ductal breast carcinoma: a case report

**DOI:** 10.1186/s12890-024-02897-y

**Published:** 2024-02-22

**Authors:** Junqing Gan, Meiyue Liu, Fei Liu, Junxiu Wen, Wenjuan Fu, Jinghao Jia

**Affiliations:** 1 Department of Chemoradiation, North China University of Science and Technology Affiliated Hospital, Tangshan, Hebei China; 2 Department of Radiotherapy, North China University of Science and Technology Affiliated Hospital, Tangshan, Hebei China; 3 Department of Pathology, North China University of Science and Technology Affiliated Hospital, Tangshan, Hebei China

**Keywords:** Synchronous double primary malignant tumor, Small cell lung cancer, Breast invasive ductal carcinoma

## Abstract

**Background:**

Although lung and breast cancers are common malignancies, the occurrence of primary synchronous neoplasms involving these organs has been rarely reported in literature.

**Case presentation:**

A 75-year-old female patient presented at a local hospital with a ten-day history of dizziness and slurred speech. A CT contrast-enhanced scan revealed a 4.2 cm mass in the lower lobe of the right lung and a 3.8 cm space-occupying lesion in the right breast. Subsequent breast ultrasound identified a hypoechoic lesion measuring5.41 × 4.75 × 3.06 cm in the right breast, and an ultrasound-guided biopsy confirmed the presence of infiltrating ductal carcinoma of the right breast. The immunohistochemistry analysis of the breast mass revealed positive staining for ER, PR, HER-2, AR and Ki67 in the tumor cells, while negative staining was observed for P63, Calponin, CK5/6 and CK14. MR imaging of the head detected abnormal signals in the right frontal lobe (3.6 cm×2.9 cm in size), left cerebellar hemisphere, and punctate enhancement in the left temporal lobe, indicating potential metastasis. Pathological examination of a lung biopsy specimen confirmed the presence of small cell lung cancer (SCLC). Furthermore, immunohistochemistry analysis of the lung lesions demonstrated positive staining for TTF-1, CK-Pan, Syn, CgA, CD56, P53 (90%) and Ki67 (70%), and negative staining for NapsinA and P40 in the tumor cells. The patient’s diagnosis of SCLC with stage cT2bN0M1c IVB and brain metastases (BM), as well as invasive ductal breast carcinoma (IDC), was confirmed based on the aforementioned results. Whereupon we proposed a treatment plan consisting of whole-brain radiation (40 Gy/20fractions), focal radiotherapy (60 Gy/20fractions), and adjuvant concurrent chemotherapy with oral etoposide (50 mg on days 1 to 20).

**Conclusions:**

To the best of our knowledge, the present case is the first of its kind to describe the synchronous double cancer, consisting of primary SCLC and IDC.

## Introduction

Multiple primary malignant tumors (MPMTs) are defined as two or more malignancies with various pathogenic origins detected simultaneously or successively in an individuality [1]. Due to the time interval of diagnosis for the first and second primary tumors, MPMTs can be stratified into synchronous (< 6 months) and metachronous (≥ 6 months) MPMTs [2]. Warren and Gates went a step further by refining diagnostic criteria for MPMTs:(1) each tumor must present malignant, (2) each tumor must be histologically distinct, (3) all tumors must be primary rather than metastases of each other [3]. Much of the increased incidence of MPMTs can be attributed to advances in technology for cancer diagnostics and treatments, which have markedly increased the survival of cancer patients [4]. In a retrospective study that included 1066 patients with breast cancer, 6 were diagnosed with synchronous breast cancer and lung cancer. Among them, 5 cases are lung adenocarcinoma cancers and 1 case is lung squamous cell carcinoma [5]. The present study reports a case of double primary cancer, comprising small cell lung cancer (SCLC), as well as invasive ductal carcinoma of breast (IDC). To our knowledge, a review of literature in PubMed revealed no case similar to ours.

## Case presentation

**Fig. 1:**
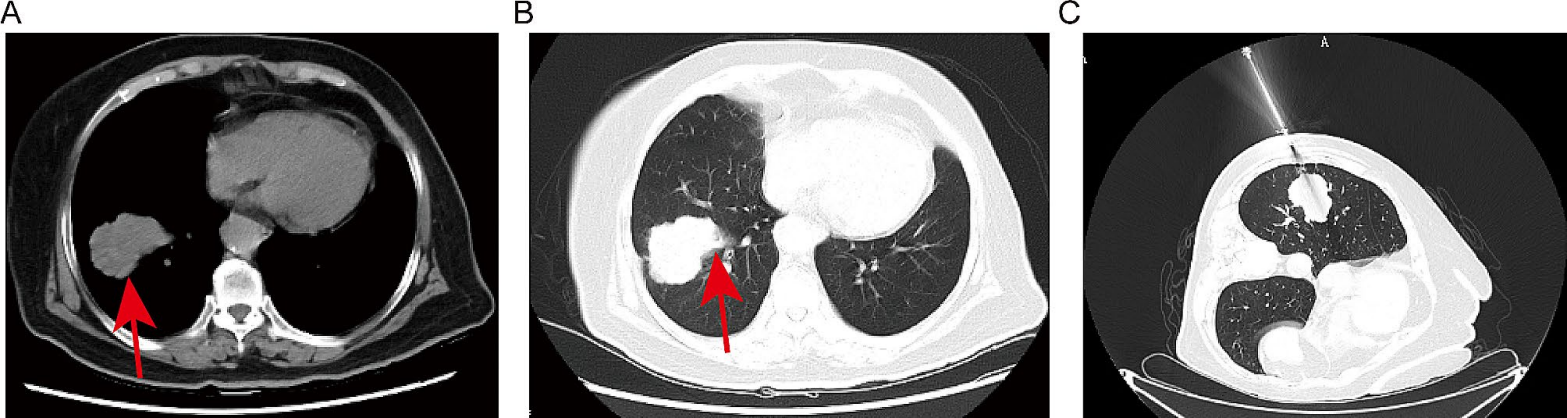
CT scan showed a tumor mass of irregular border in the lower lobe of the right lung. (**A**) Mediastinal window and (**B**) lung window showed a right-side lung mass (arrow indicated the location of lesion); (**C**) The localization needle inserted into the chest wall indicated the needle entry route

**Fig. 2:**
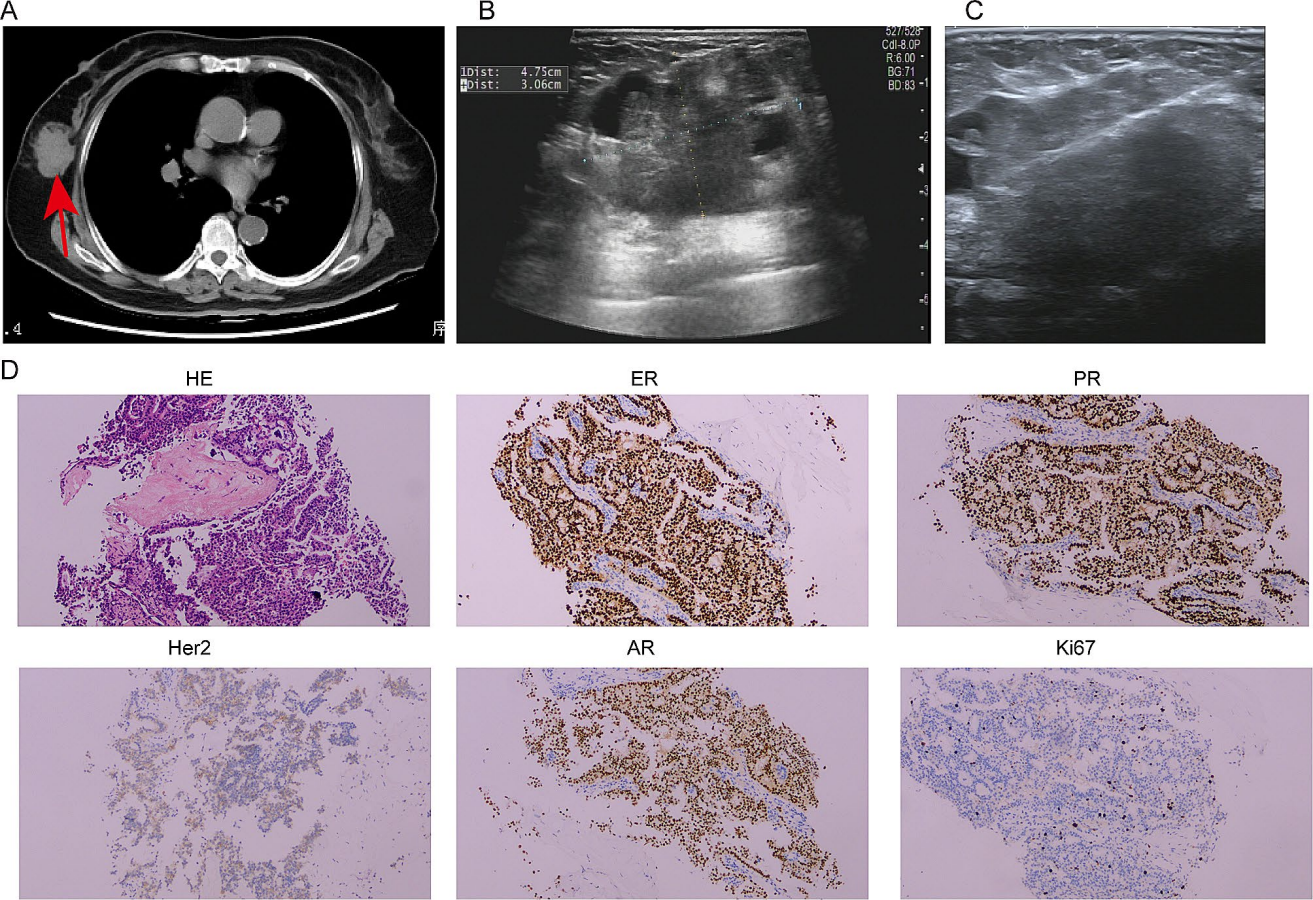
Lesion in the breast. (**A**) CT scan and (**B**) breast ultrasound showed a tumor mass in right breast; (**C**) An ultrasound guided fine needle aspiration (FNA) was performed; (**D**) Representative HE and IHC positive staining of the primary lesion in right breast. Arrow indicated the location of lesion

**Fig. 3:**
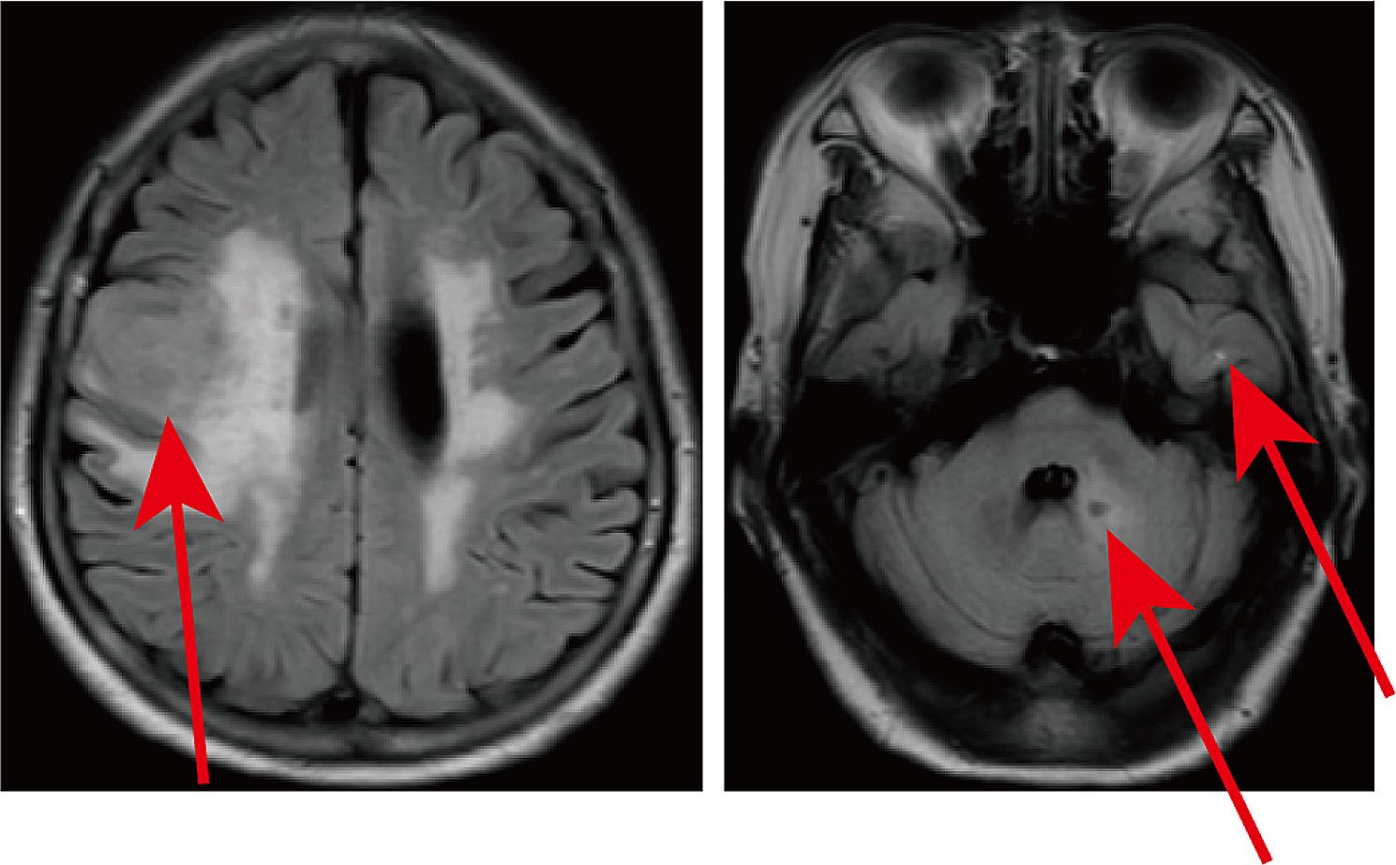
MR demonstrated abnormal signals in the right frontal lobe, left cerebellar hemisphere, and punctate enhancement in the left temporal lobe. Arrow indicated the location of lesion

**Fig. 4:**
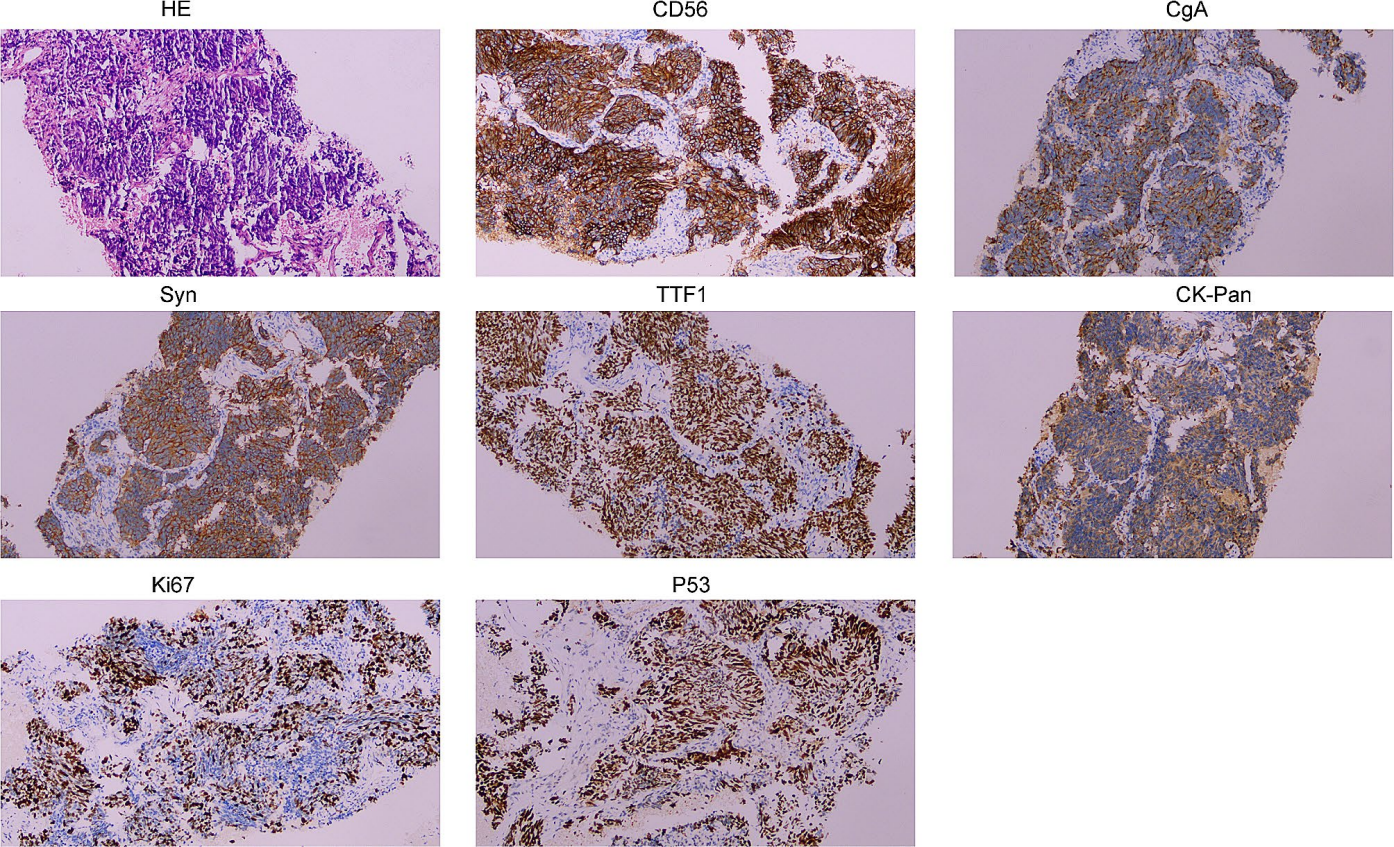
Representative HE and IHC positive staining of the primary lesion in right lung

**Fig. 5:**
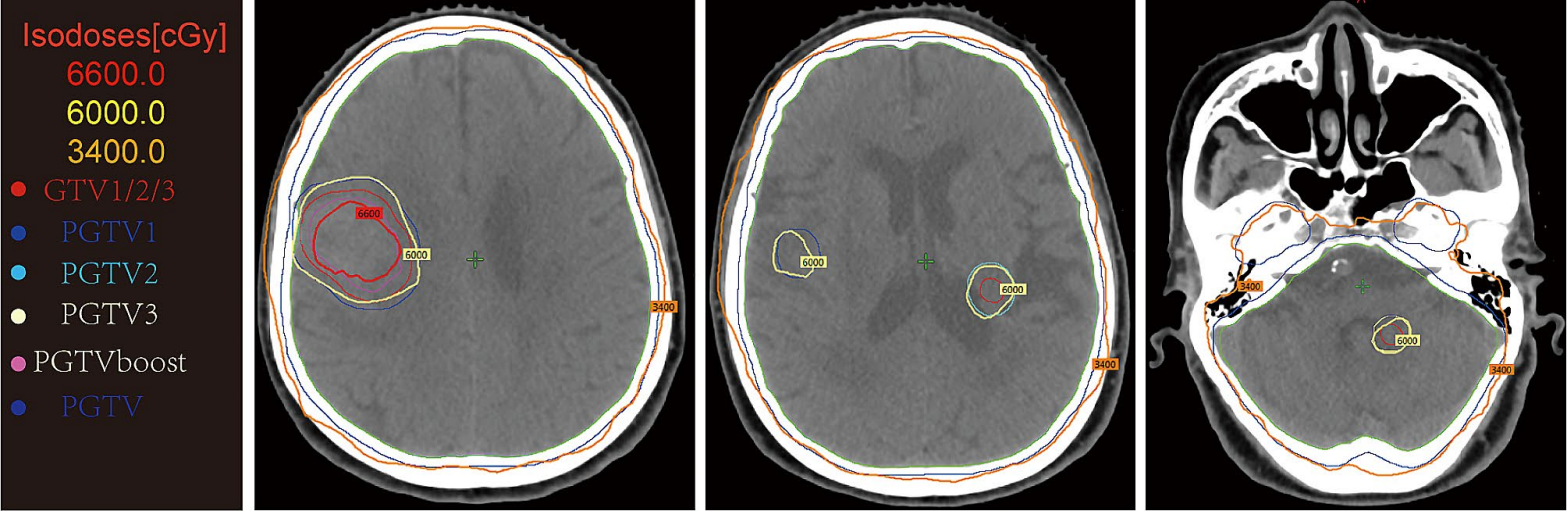
The location of brain tumors from positioning CT images and final excellent dose distribution for each tumor

A 75-year-old female patient visited to local hospital with dizziness, slurred speech for 10 days, and then was found to have a space-occupying lesion in right lung by chest CT scanning. For further treatment, the patient was referred to our institution. She lost 5 kg within 10 days, did not smoke and had no family history of malignancy. Physical examination revealed a 5 cm×5 cm mass with tough texture and indistinct borders in right mammary area, while the left nipple and breast showed no abnormal findings grossly. Other systemic examinations were unremarkable. Her past medical history included ten years of cerebrovascular disease and five years of hypertension and coronary heart disease. The vital parameters exhibited values within the established normal range. Laboratory data disclosed complete blood count, liver and kidney function tests were within normal limits. The tumor marker showed raised NSE and CEA while other markers were within normal range (Table 1). CT contrast-enhanced scan showed a mass 4.2 cm in diameter in the lower lobe of the right lung (Fig. 1A-B) and a 3.8 cm space-occupying lesion in right-sided breast (Fig. 2A). The patient underwent CT-guided percutaneous biopsy in the right lung neoplasm (Fig. 1C) and it took a few days to achieve results. Breast ultrasound revealed the presence of 5.41 × 4.75 × 3.06 cm hypoechoic lesion in the right breast and mammary duct ectasia, furthermore, no obvious lymph node enlargement was detected in the two axillary fossa, subclavicular regions and parasternal (Fig. 2B). The histopathological examination of the ultrasound-guided biopsy (Fig. 2C) revealed the presence of tumor cells organized in sheets and nests, exhibiting scant cytoplasm. The nuclei of these cells appeared round or oval, containing granular stippled chromatin and visible nuclear divisions. Notably, no apparent nucleoli were observed, and areas of necrosis were evident. Immunohistochemistry result for breast mass testified strongly positive staining for estrogen receptor (ER) and progesterone receptor (PR) in most tumor cells (3+), 2 + staining for human epithelial receptor 2 (HER-2), 3 + staining for androgen receptor (AR) and 10% positive Ki67 in tumor cells. P63, Calponin, CK5/6 and CK14 was negative in tumor cells (Fig. 2D). Based on the pathological and the immunohistochemical findings, the lesion was regarded as invasive ductal carcinoma (IDC) of the right breast. It was recommended that the patient underwent fluorescence in situ hybridization (FISH) to detect HER-2 status, however, the patient refused the procedure. MR imaging of the head showed abnormal signals in the right frontal lobe (3.6 × 2.9 cm in size), left cerebellar hemisphere, and punctate enhancement in the left temporal lobe, which should be considered for metastasis (Fig. 3). Based on these findings, a provisional diagnosis of IDC with brain metastasis (BM) was made. To alleviate the symptoms, brain focal radiotherapy was delivered. Due to larger right frontal lobe lesion, tumor cells—especially those located in the center of this lesion—often face a severe microenvironment lacking oxygen. Thus, she underwent brain focal radiotherapy, simultaneously high-dose radiotherapy in the center of the foci and prescribed dose was 95%PGTVboost (the center of lesion in right frontal lobe) 66 Gy/3.3 Gy/20fractions; 95%PGTV1(lesion in right frontal lobe) 60 Gy/3Gy/20fractions; 95%PGTV2(lesion in left temporal lobe) 60 Gy/3Gy/20fractions; 95%PGTV3(lesion in left cerebellar hemisphere) 60 Gy/3Gy/20fractions. After 3 days of radiotherapy, pathological findings presented that lung lesions cells were distributed in flaky nest-like shape, arranged densely, with hyperchromatic nuclei, visible areas of focal necrosis, moreover, immunohistochemistry of lung lesions showed positive staining for thyroid transcription factor-1(TTF-1), cytokeratin-PAN (CK-Pan), synaptophysin (Syn), chromogranin A (CgA), CD56, P53 (90%) and Ki67 (70%), and negative staining for NapsinA and P40 in tumor cells (Fig. 4). The findings on pathology and immunohistochemistry suggested SCLC. After discussion, the final diagnosis was SCLC (cT2bN0M1c IVB) with BM, IDC. Whereupon we offered to treat patient with whole-brain radiation (40 Gy/20fractions) and focal radiotherapy (60 Gy/20fractions) and adjuvant concurrent chemotherapy with oral etoposide (50 mg d1-d20). Regretfully, the effect was not observed as the patient refused further therapy and follow-up. Completed dose was 95%PGTVboost 66 Gy/3.3 Gy/20fractions; 95%PGTV1 60 Gy/3Gy/20fractions; 95%PGTV2 60 Gy/3Gy/20fractions; 95%PGTV3 60 Gy/3Gy/20fractions; 95%PTV 34 Gy/2Gy/17fractions (Fig. 5).

**Table 1:** Laboratory data of tumor markers

Laboratory test	Value	Unit	Reference range
AFP	2.860	ng/ml	0–7
CA125	18.580	U/mL	0–35
CA153	7.810	U/mL	0–25
CA199	9.540	U/mL	0–39
CA724	3.980	U/mL	0-6.9
CEA	**5.590**↑	ng/ml	0-3.4
HCG	1.290	mIU/mL	0–3
NSE	**28.430**↑	μg/ml	0-15.2
SCC	0.856	ng/ml	0.5–2.7

Note: the arrows indicate the elevated expression

## Discussion and conclusions

Both female breast cancer and lung cancer are highly common malignancies around the world, ranking as the top two cancers in terms of incidence in patients [6]. IDC is the most common type of breast cancer which accounts for 75% of all cases [7]. Furthermore, approximately 15% of lung cancers are SCLC [8]. Wu et al. used the next generation sequencing to detect simultaneous primary lung adenocarcinoma and breast cancer [9]. However, simultaneous detection of SCLC and IDC represents an uncommon event, despite the increasing overall incidence of multiple primary malignant tumors. We present a case of synchronus double primary SCLC and IDC in an old female. To date, no similar case has been reported literature. In the diagnosis of double primary carcinoma, the possibility of tumor metastasis should be excluded. In breast cancer patients with metastatic disease, lung is the common site of metastasis [10]. Moreover, Ali et al. identified 16 metastatic lung tumors to the breast, among which 12 non-small cell lung cancer,1 large-cell neuroendocrine, 1 atypical carcinoid and 2 small-cell carcinomas. Zhao et al. demonstrated a male SCLC with breast mass as the first manifestation [11]. In a number of cases, it is difficult to differentiate between two primary neoplasms or metastatic diseases. In this case study, the malignant features of each tumor were synchronously confirmed by pathological and immunohistochemical examination.

A retrospective cohort study of metachronous second primary cancers demonstrated that uterus, ovary and thyroid were the most frequent sites for developing a second primary cancer after first breast cancer, moreover, thyroid, larynx, mouth/pharynx were the most frequent sites for developing a second primary cancer after first lung cancer [12]. However, a 20-year study verified that lung cancer patients were at higher risk of oesophageal and head and neck cancers comparing to other residents from Queensland [13]. As showed, no exact correlation for predilection site was noted between double primary malignant tumors. Therefore, the possibility of double or multiple primaries should be taken into account when treating patients with multiple tumors.

BM most commonly occur in patients with cancers from the lung and breast, have poor prognosis and high mortality rate, and lack of effective treatment [14]. According to the statistics, BM are a frequent complication in lung cancer patients, presenting in approximately 40% of patients with advanced adenocarcinoma and 50% with SCLC [15]. BM are present at diagnosis in approximately 18% of SCLC at initial diagnosis, and can reach 50–65% of cases within two years [16], moreover, the median survival of SCLC with BM is only 4.9 months [17]. The incidence of BM from breast cancer still following the lung cancer ranks second, accounting for about 10 to 30% [18], and with a median time of BM occurrence 2–3 years after the initial breast cancer diagnosis [19]. SCLC is distinguished by its small size, limited adhesion, and tendency to spread early, resulting in the formation of small primary lesions and significant metastatic lesions [20, 21]. In this particular case, a higher level of ki67 expression (70%) suggests a heightened degree of malignancy within the tumors. Additionally, MR imaging reveals the presence of multiple brain metastases, with larger instances measuring up to 3.6 × 2.9 cm. Shi et al. found that cerebellar hemisphere was a high-risk brain region in the SCLC [22]. Breast ultrasound revealed no obvious lymph node enlargement was detected in the two axillary fossa, subclavicular regions and parasternal. Immunohistochemistry result for breast mass testified strongly positive staining for ER and PR in most tumor cells (3+) and 10% positive Ki67 in tumor cells. The biomarker Ki67 is routinely used for assessing the proliferative index of primary breast cancer tissue and is the single most important prognostic factor for breast cancer brain metastasis [23]. In summary, we consider that the patient has a high possibility of SCLC with BM. The final diagnosis was SCLC (cT2bN0M1c IVB) with BM, IDC.

Currently, no clear and unified clinical treatment guideline for synchronous primary cancers has been developed [17]. Surgery remains the primary treatment option [24]. However, palliative care was ultimately implemented due to the following factors: Firstly, the patient presented a medical history encompassing hypertension, coronary heart disease, and cerebrovascular disease, alongside current symptoms of dizziness and slurred speech, rendering them unsuitable for surgical intervention. Secondly, MR findings indicated the presence of multiple brain metastases and a suboptimal response to dehydration treatment. Lastly, the patient explicitly declined surgical intervention. The aims of palliative therapy were to control local tumor growth and ease and improve symptoms, while improving and preserving the patient’s quality of life. We finally formulated whole-brain radiation (40 Gy/20fractions) and focal radiotherapy (60 Gy/20fractions) and adjuvant concurrent chemotherapy with oral etoposide treatment plan due to following reasons:1) many breast cancers had a slow disease progression and relatively good prognosis [25]; however, SCLC is life-threatening due to its rapid progression [26]. So, the chemotherapy that targets the two tumors and concentrates on the most aggressive seems the most reasonable treatment.2) First-line standard chemotherapy for patients with SCLC is a combination of etoposide with platinum [27]. However, patient may be unable to tolerate intensive chemotherapy due to severe dizziness. Furthermore, the compliance of patients is relatively poor. Meanwhile, etoposide is chemotherapeutic agents extensively used to treat a wide spectrum of solid tumors (including breast cancer) [28]. In addition, oral etoposide increases the sensitivity of tumor cells to radiation therapy [29]. Single-agent etoposide is thus considered a most suited for treatment option.3) Before lung biopsy pathology were reported, we initially diagnosed breast cancer with BM, so focal radiotherapy was given to alleviate dizziness. Due to larger right frontal lobe lesion, tumor cells—especially those located in the center of this lesion—often face a severe microenvironment lacking oxygen. Thus, she underwent brain focal radiotherapy, simultaneously high-dose radiotherapy in the center of the foci. When lung biopsy pathology later confirmed primary SCLC, the diagnosis was revised to SCLC with BM. Whole-brain radiotherapy (WBRT) has remained the standard of care for patients with BM from SCLC [30]. Therefore, we finally proposed a treatment plan of whole-brain radiation and focal radiotherapy.

In summary, this manuscript was reported about an extremely case of simultaneous double primary SCLC and breast cancer. Although the case is very rare, when imaging examination reveals a mass in other organs, the possibility of a new primary tumor rather a metastase of the initial primary tumor for these patients must be seriously considered. The combination of tumor markers analysis, imaging findings and clinical characteristics may be helpful to determine an accurate preoperative diagnosis, but the final diagnosis should be dependent on the pathological and immunohistochemical examination. Given that it is a rare disease, consensus on effective therapy is unavailable and remains to be further investigated.

## Data Availability

All data generated or analysed during this study are included in this published article.

## Abbreviations

AR: Androgen receptor
BM: Brain metastases
CgA: Chromogranin A
CK-Pan: Cytokeratin-Pan
CT: Computed tomography
ER: Estrogen receptor
FISH: Fluorescence in situ hybridization
HER-2: Human epithelial receptor 2
IDC: Invasive ductal carcinoma
MPMTs: Multiple primary malignant tumors
PR: Progesterone receptor
SCLC: Small cell lung cancer
Syn: Synaptophysin
TTF-1: Thyroid transcription factor-1
